# An Excellent Appointment

**Published:** 1881-01

**Authors:** 


### AN EXCELLENT APPOINTMENT.

Dr. Andrews, the Superintendent of the Buffalo State Asylum for the Insane, has wisely chosen for his First Assistant Physician, our talented young friend and townsman, Dr. Wm. D. Granger. The appointment secured the endorsement of the most influential members of the profession in this city, and is a most excellent selection for the interests of the institution, and for the special work it seeks to advance. Inasmuch as the Journal, in the interests of the profession and the institution, heartily and earnestly seconded this selection, we feel that our congratulations are due not only to the successful appointee, but to the profession, of which he is an ambitious and energetic worker.

In this connection we beg to state, for the information of the public, that the Asylum has already received a considerable number of patients, and with its admirable facilities and healthy location will fully maintain the foremost position it aims to secure among the public institutions of the Empire State.

